# The miRFIB-Score: A Serological miRNA-Based Scoring Algorithm for the Diagnosis of Significant Liver Fibrosis

**DOI:** 10.3390/cells8091003

**Published:** 2019-08-29

**Authors:** Joeri Lambrecht, Stefaan Verhulst, Hendrik Reynaert, Leo A. van Grunsven

**Affiliations:** 1 Department of Basic (Bio-)Medical Sciences, Liver Cell Biology Research Group, Vrije Universiteit Brussel, 1050 Brussels, Belgium; 2 Department of Gastroenterology and Hepatology, University Hospital Brussels (UZ Brussel), B-1090 Brussels, Belgium

**Keywords:** biomarker, NAFLD, viral liver disease, alcoholic liver disease, microRNA, hepatic stellate cell, fibrosis, diagnosis, liquid biopsy

## Abstract

**Background**: The current diagnosis of early-stage liver fibrosis often relies on a serological or imaging-based evaluation of the stage of fibrosis, sometimes followed by an invasive liver biopsy procedure. Novel non-invasive experimental diagnostic tools are often based on markers of hepatocyte damage, or changes in liver stiffness and architecture, which are late-stage characteristics of fibrosis progression, making them unsuitable for the diagnosis of early-stage liver fibrosis. miRNAs control hepatic stellate cell (HSC) activation and are proposed as relevant diagnostic markers. **Methods**: We investigated the possibility of circulating miRNAs, which we found to be dysregulated upon HSC activation, to mark the presence of significant liver fibrosis (F ≥ 2) in patients with chronic alcohol abuse, chronic viral infection (HBV/HCV), and non-alcoholic fatty liver disease (NAFLD). **Results**: miRNA-profiling identified miRNA-451a, miRNA-142-5p, Let-7f-5p, and miRNA-378a-3p to be significantly dysregulated upon in vitro HSC activation, and to be highly enriched in their extracellular vesicles, suggesting their potential use as biomarkers. Analysis of the plasma of patients with significant liver fibrosis (F ≥ 2) and no or mild fibrosis (F = 0–1), using miRNA-122-5p and miRNA-29a-3p as positive control, found miRNA-451a, miRNA-142-5p, and Let-7f-5p, but not miRNA-378a-3p, able to distinguish between the two patient populations. Using logistic regression analysis, combining all five dysregulated circulating miRNAs, we created the miRFIB-score with a predictive value superior to the clinical scores Fibrosis-4 (Fib-4), aspartate aminotransferase/alanine aminotransferase (AST/ALT) ratio, and AST to platelet ratio index (APRI). The combination of the miRFIB-score with circulating PDGFRβ-levels further increased the predictive capacity for the diagnosis of significant liver fibrosis. **Conclusions**: The miRFIB- and miRFIB^p^-scores are accurate tools for the diagnosis of significant liver fibrosis in a heterogeneous patient population.

## 1. Introduction

Liver fibrosis and subsequent cirrhosis result in over one million deaths per year worldwide, making it the eleventh most common cause of death in adults [1]. The three most important causes for the development of liver fibrosis are chronic alcohol abuse, chronic infection with the hepatitis B (HBV) or C (HCV) virus, and metabolic syndrome, which can result in non-alcoholic fatty liver disease (NAFLD) and non-alcoholic steatohepatitis (NASH) [2]. Upon the chronic presence of such liver injury-causing circumstances, inflammatory signals within the liver will induce hepatocyte-damage and the activation of liver-resident hepatic stellate cells (HSCs) towards a myofibroblastic phenotype. This activation process is marked by an excessive production and deposition of extracellular matrix [3]. The early development of fibrosis, and its progression towards cirrhosis, can be halted and even reverted upon suitable treatment, such as the administration of anti-viral drugs, or significant life-style changes [4]. Importantly, overall disease outcome improves when the treatment is started as early as possible in the disease process [5].

To date, liver biopsy remains the gold standard for grading and staging liver fibrosis. Unfortunately, this technique is associated with sampling and interpretation variability [6], doubtable cost–benefit ratios [7], and a risk of pain and bleeding [8]. These drawbacks limit the use of liver biopsy as a tool for screening or follow-up. Various non-invasive diagnostic tools have been developed, of which some have made their way into clinical practice, including serological scoring tools, such as the enhanced liver fibrosis (ELF) test, aspartate aminotransferase/alanine aminotransferase (AST/ALT) ratio, Fibrosis-4 (Fib-4) score, and the AST to platelet ratio index (APRI), and imaging-based tools, such as transient elastography (FibroScan^®^), shear wave elastography (SWE), and acoustic radiation force impulse (ARFI) [9]. However, these non-invasive techniques have not yet led to full redundancy of the liver biopsy, especially due to their limited sensitivity and specificity for the detection of early stages of liver fibrosis.

MicroRNAs (miRNAs) are non-coding, single-stranded RNA structures of approximately 22 nucleotides long, and function as posttranscriptional gene regulators. More specifically, through binding to the 3’ untranslated regions of target messenger RNAs (mRNA), they can induce their cleavage, or prevent their translation into proteins [10]. Some miRNAs are known to be expressed in a cell- or tissue-specific manner. miRNAs are found in almost all body fluids, where they obtain stability by packaging into extracellular vesicles, or association to Argonaute2 or high-density lipoproteins [11]. Recent research has identified the potential of miRNAs to be used as diagnostic tools for specific subsets of liver disease, often focusing on the diagnosis of liver cirrhosis and hepatocellular carcinoma (HCC) [12,13]. However, the diagnostic value of individual miRNAs, or miRNA-panels, for the identification of early stages of liver fibrosis in heterogeneous patient populations remains to be proven.

In this study, we aimed to investigate the diagnostic value of miRNAs for the identification of significant liver fibrosis in a heterogeneous patient cohort suffering from chronic liver disease. We found that plasma levels of several individual miRNAs associated with HSC activation can distinguish between no or mild fibrosis (F0–1) and significant liver fibrosis (F ≥ 2). More importantly, the combination of the plasma levels of five miRNAs and PDGFRβ protein levels into the miRFIB^P^-score increased the predictive capacity for the diagnosis of significant liver fibrosis and outperformed clinical scores, such as Fib-4, the AST/ALT ratio, and APRI.

## 2. Materials and Methods

### 2.1. Animal Studies

The use and care of animals was reviewed and approved by the Ethical Committee of Animal Experimentation of the Vrije Universiteit Brussel (Brussels, Belgium) in project 16-212-2, and was carried out in accordance with European Guidelines for the Care and Use of Laboratory Animals. All mice were housed in a controlled environment with free access to water and food. Primary HSCs were isolated from male Balb/c mice aged 25 to 30 weeks (Charles River Laboratories, L’Arbresle, France), as described earlier [14,15]. Briefly, murine livers were digested by enzymatic solutions consisting of collagenase (Roche diagnostics, Mannheim, Germany) and pronase E (Merck, Darmstadt, Germany). The resulting cell suspension was centrifuged at low speed to remove hepatocytes. Hepatic stellate cells (HSCs) were purified from the non-parenchymal fraction based on their buoyancy, using an 8% Nycodenz (Axis-shield PoC AS, Dundee, Scotland) solution. Isolated HSCs were cultured on regular tissue culture dishes (Greiner Bio-One, Vilvoorde, Belgium), in Dulbecco’s modified Eagle’s medium (Lonza, Verviers, Belgium) supplemented with 10% foetal bovine serum (Lonza, Verviers, Belgium), 2 mM L-glutamine (Ultraglutamine 1^®^) (Lonza), 100 U/mL penicillin, and 100 μg/mL streptomycin (Pen-Strep^®^) (Lonza), inducing in vitro myofibroblastic transdifferentiation. Cell purity was confirmed by the presence of lipid droplets and staining for HSC-specific markers.

For the in vivo induction of liver fibrosis, 10-week old mice received eight intraperitoneal injections of 15 µL carbon tetrachloride (CCl_4_) diluted in 85µL mineral oil (Sigma-Aldrich, St. Louis, MO, USA) per 30 g bodyweight over a period of four weeks. Mice were sacrificed 24 h after the last injection.

### 2.2. Patient Cohort

Patients with liver fibrosis caused by chronic alcohol abuse and chronic viral hepatitis were recruited from the Department of Gastroenterology and Hepatology of the University Hospital of Brussels (UZ Brussel), Belgium. The extent of liver fibrosis in these patients was determined based on transient elastography (FibroScan^®^, Echosens, France). Patients with at least 10 valid stiffness measurements with a success rate of minimum 60% were included in the final analysis. Cut-off values used to discriminate fibrotic stages equal to or more than F2, F3, and F4 were taken at 7.2 kPa, 9.5 kPa, and 12.5 kPa [16], respectively. Patients with liver fibrosis suffering from NAFLD were recruited from the Diabetes Centre of the University Hospital of Brussels (UZ Brussel, Brussels, Belgium) in collaboration with the Department of Gastroenterology and Hepatology (UZ Brussel, Brussels, Belgium). In these patients, the extent of fibrosis was determined by use of acoustic radiation force impulse (ARFI), using cut-off values of 1.25 m/s, 1.54 m/s, and 1.84 m/s to identify a fibrotic stage equal to, or more than F2, F3, and F4, respectively. All NAFLD-patients had Diabetes Mellitus type 2. The protocol of this study was approved by the local ethical committee of the UZ Brussel and Vrije Universiteit Brussel (reference number 2015/297; B.U.N. 143201525482) and was in accordance with the Declaration of Helsinki. An informed consent was obtained from all participants, prior to inclusion in the study.

### 2.3. Blood Collection

Blood samples were collected by venepuncture into evacuated ethylenediaminetetraacetic acid (EDTA-KE) S-Monovette tubes (Sarstedt AG & Co, Nümbrecht, Germany) on the day of FibroScan or ARFI. All samples were subjected to haematological and biochemical analyses. Plasma was created within a maximum timespan of 2 h after collection, using a two-step centrifugation protocol of 1500 g for 10 min (4 °C), followed by 2000 g for 3 min (4 °C). Plasma was frozen at −80 °C until further use.

### 2.4. Serological Scoring of Fibrosis

Haematological analyses and subsequent serological scoring algorithms, such as our recently developed PRTA-score [17] and the clinical algorithms Fib-4, APRI, and the AST/ALT ratio, were used to validate the elastography-based fibrosis scoring. The PRTA-score, Fib-4, and APRI were calculated using following formulae:

Fib-4 = age × AST[IU/L]/(thrombocytes[10^9^/L] × (ALT[IU/L])^1/2^);

APRI = (AST[IU/L]/ULN)/thrombocytes[10^9^/L];

PRTA-score = (sPDGFRβ[pg/mL] × 100)/(albumin[g/L] × (thrombocytes[/mm^3^]/100)).

### 2.5. Messenger RNA and microRNA Analysis

miRNAs were extracted from 500 µL human- or 150 µL mouse-plasma by use of the Nucleospin^®^ miRNA Plasma kit (Macherey-Nagel, Düren, Germany), using the manufacturer’s protocol. Caenorhabditis elegans miRNA-39 (Cel-miRNA-39) (Qiagen, Hilden, Germany) was added into the plasma lysate before the start of the extraction protocol and served as an external processing control. Total RNA from liver tissue and cultured hepatic stellate cells was extracted by the TRIzol reagent (ThermoFisher scientific, Waltham, USA) and ReliaPrep^TM^ RNA Miniprep system (Promega, Madison, WI, USA), using the manufacturers’ protocol. Messenger RNA (mRNA) was reverse-transcribed into complementary DNA (cDNA) using a mixture of hexamer random primers, Moloney murine leukemia virus reverse transcriptase (M-MLV RT) Buffer, deoxyribonucleotide triphosphate (dNTP) mix, M-MLV RT RNase (H-) Point mutant, and RNasin^®^ Plus RNase Inhibitor (Promega). MicroRNA (miRNA) was reverse transcribed into cDNA using the miScript II RT kit (Qiagen, Hilden, Germany). The expression profiles of selected mRNA and miRNAs were analyzed by quantitative real-time polymerase chain reaction (qPCR), using the GoTaq qPCR Master Mix with BRYT green (Promega) and the miScript SYBR Green PCR kit (Qiagen), respectively, in the QuantStudio 3 real-time PCR system (ThermoFisher scientific). Obtained results were analyzed using the QuantStudio 3 Design and Analysis Software (ThermoFisher scientific). Individual gene and miRNA expression was normalized to Gapdh, RNU6, or Cel-miRNA-39, as appropriate. Relative expression was calculated using the comparative Ct method (2^−ΔΔCT^). miRNA- (Supplementary Table S1) and gene- (Supplementary Table S2) specific primers were produced by Integrated DNA Technologies (IDT, Leuven, Belgium).

### 2.6. Histological Evaluation

Four-micrometer paraffin-embedded liver tissue sections were cut, deparaffinized, and rehydrated before staining with Sirius Red/Fast Green or Hematoxyline/Eosin. The sections were then washed, dehydrated, and mounted with DPX mounting medium. Whole slide images were taken using the Aperio CS2 image capture device (Leica, Diegem, Belgium). Collagen staining was quantified using the Orbit Image Analysis software (Actelion Pharmaceuticals Ltd, Allschwil, Switzerland) [18].

### 2.7. Immunocytochemistry

After the isolation of primary murine hepatic stellate cells, the cells were cultured on coverslips for 24 h or 10 days. Cells were washed with PBS and fixed with formalin for 10 min. The cells were then washed three times with PBS and stored at 4 °C until further use. Prior to staining, the cells were permeabilized using PBS supplemented with 0.1% Triton-X (3 × 5 min). Afterwards, cells were incubated for 30 min with 0.1% Triton-X PBS containing 2% bovine serum albumin (BSA), to block non-specific binding sites. The cells were incubated overnight at 4 °C with anti-Desmin (1:200, RB-9014-P, ThermoFisher scientific), or anti-Vimentin (1:200, V5255, Sigma-Aldrich). Three wash-steps using 0.1% Triton-X PBS were applied, followed by incubation with donkey anti-rabbit Alexa Fluor 488 secondary antibody (1:200, A21206, ThermoFisher scientific), donkey anti-mouse Alexa Fluor 488 secondary antibody (1:200, A21202, ThermoFisher scientific), or Cy3-coupled mouse anti-αSMA primary antibody (1:100, C6198, Sigma-Aldrich) for 1.5 h. Coverslips were mounted with 4′,6-diamidino-2-phenylindole (DAPI)-containing mounting medium (Dako, Denmark). Images were taken using the EVOS FL fluorescence microscope (ThermoFisher).

### 2.8. Statistical Analysis

Data were analyzed using GraphPad Prism 8 (GraphPad, Palo Alto, USA). Quantitative variables are expressed as means ± standard error of the mean (SEM) or expressed as boxplots using the Tukey representation. Statistical analyses were performed using the Student’s t-test, Mann–Whitney test, and One-Way ANOVA with Dunnett post hoc test, as appropriate. Categorical values were analyzed using the Chi-square test. The diagnostic accuracy and performance of the mentioned miRNAs and serological scores were determined using receiver operating characteristics (ROC) curves, and the area under the curve (AUC) was calculated. Sensitivity and specificity were calculated based on the highest Youden’s index values [19]. Correlation studies were executed using the Spearman’s correlation test. Logistic regression analyses were performed using MedCalc version 18 (MedCalc Software, Ostend, Belgium). The sufficiency of the sample size was confirmed by MedCalc version 18 using in house preliminary results and a type I error rate (α) of 5% and a power (1-β) of 80%. Results were considered statistically significant when *p* < 0.05.

## 3. Results

### 3.1. Identification of Candidate HSC-Linked miRNAs

**Figure 1:**
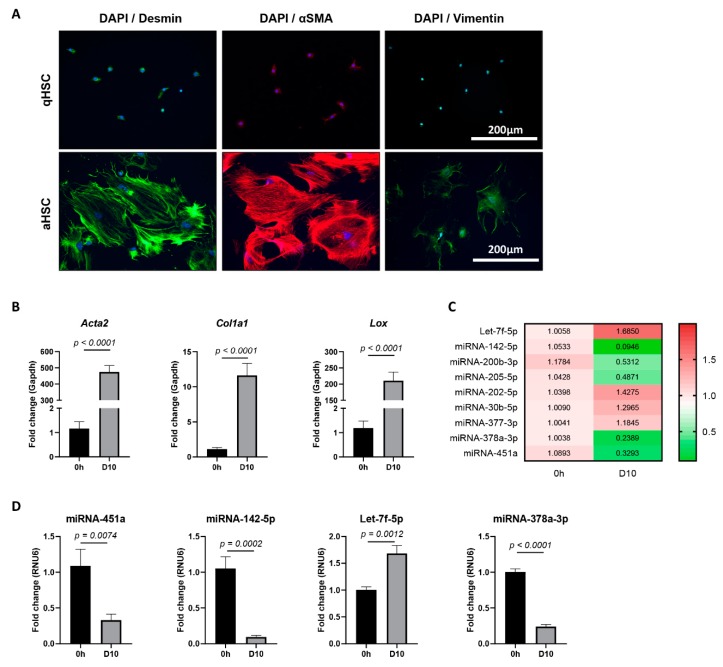
miRNA expression in mouse in vitro activated hepatic stellate cells (HSCs). (**A**) Immunofluorescence staining of quiescent (24 h of culture) and activated (10 days of culture) primary mouse HSCs for activation markers Desmin, αSMA, and Vimentin. 4′,6-Diamidino-2-phenylindole (DAPI) was used as nuclear staining. Representative images are shown. (**B**) mRNA expression levels determined by quantitative polymerase chain reaction (qPCR) of HSC-activation markers *Acta2*, *Col1a1*, and *Lox* in freshly isolated HSCs (0 h), as compared to HSCs activated by 10 days of culture (D10). (**C**) Heatmap of relative expression levels, as determined by qPCR, for selected candidate miRNAs in activated HSCs (D10), as compared to freshly isolated HSCs (0 h). (**D**) miRNA-451a, miRNA-142-5p, Let-7f-5p, and miRNA-378a-3p were found to be significantly dysregulated upon HSC activation. One-tailed unpaired t-test analysis was used to determine statistical significance. Results are shown as mean ± standard error of the mean (SEM); *n* = 5.

As hepatic stellate cell (HSC) activation is an early event of liver fibrosis initiation and progression, we hypothesized that HSC-derived circulating miRNAs could be suitable markers for early stage liver fibrosis. In order to identify candidate miRNAs, NanoString analysis was performed on extracellular vesicles (EVs), both microvesicles and small extracellular vesicles (sEV), obtained from the conditioned medium of in vitro activating primary murine HSCs. To this end, primary mouse HSCs were plated on plastic tissue culture dishes for 10 days. The activation of cultured HSCs was verified on a protein level by the up-regulation of HSC-activation markers Desmin, α-SMA, and Vimentin (Figure 1A), and on an mRNA level by *Acta2*, *Col1a1*, and *Lox* (Figure 1B). Although the obtained miRNA counts by NanoString analysis were insufficient to compare between the quiescent and activated conditions, several miRNAs were found to be highly enriched in such EVs, as compared to the average expression level of all tested miRNAs. This list of highly shed miRNAs, and thus potential fibrosis markers, was further restricted to the miRNAs that are conserved among mouse and human, to ensure translational value, ending up with a list of nine candidate miRNAs (Supplementary Figure S1). The expression levels of these miRNAs were analyzed in the in vitro activated primary mouse HSC cultures. Expression analysis of all nine candidate miRNAs was performed by using qPCR on the cell lysate of activated HSCs, as compared to freshly isolated HSCs (Figure 1C), and identified the significant dysregulation of four miRNAs: miRNA-451a, miRNA-142-5p, Let-7f-5p, and miRNA-378a-3p (Figure 1D).

### 3.2. Identification of Ankrd52, Clcn5 and Peg10 as Potential Target Genes

**Figure 2:**
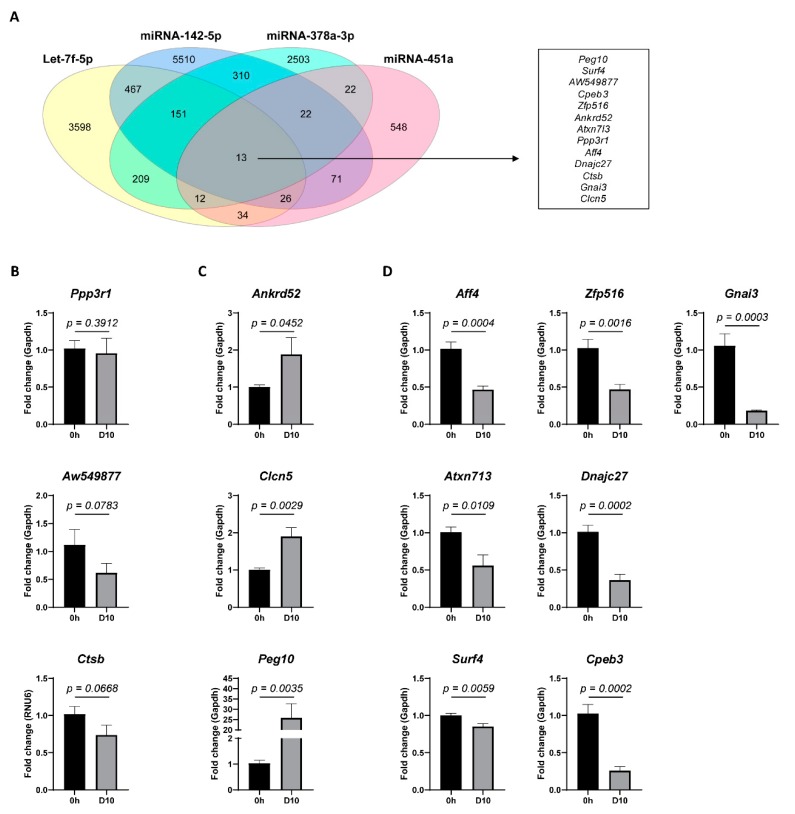
miRNA target prediction. Bioinformatics-based target prediction was carried out, using four different predictive algorithms: TargetScan, miRDB, starBase, and miRTarBase. (**A**) Venn diagram showing putative target genes for the differentially expressed miRNAs in culture activated primary mouse HSCs. A list of target genes mutual among all four miRNAs is shown. mRNA expression analysis of the identified mutual target genes in activated (D10) versus quiescent (0 h) HSCs identified (**B**) three genes with no significant difference, (**C**) three genes to be significantly up-regulated, and (**D**) seven genes to be significantly down-regulated. One-tailed unpaired t-test analysis was used to determine statistical significance. Results are shown as mean ± SEM; *n* = 5.

To investigate a potential function of miRNA-451a, miRNA-142-5p, Let-7f-5p, and miRNA-378a-3p in the HSC-activation process, a bioinformatics-based target prediction was carried out, using four different predictive algorithms: TargetScan, miRDB, starBase, and miRTarBase. Of all putative targets, thirteen genes are suggested to have all four miRNAs as post-transcriptional regulators (Figure 2A). The analysis of mRNA expression in activated HSCs, compared to freshly isolated quiescent HSCs, identified three genes that remained stable during the activation process (Figure 2B), three genes that were up-regulated (Figure 2C), and seven genes that were down-regulated (Figure 2D) upon HSC-activation. The genes that were up-regulated upon HSC activation, *Ankrd52*, *Clcn5*, and *Peg10*, are of particular interest, as miRNAs are known to regulate gene expression in a dominantly negative manner.

### 3.3. miRNA Expression Analysis in the CCl_4_-Mouse Model

**Figure 3:**
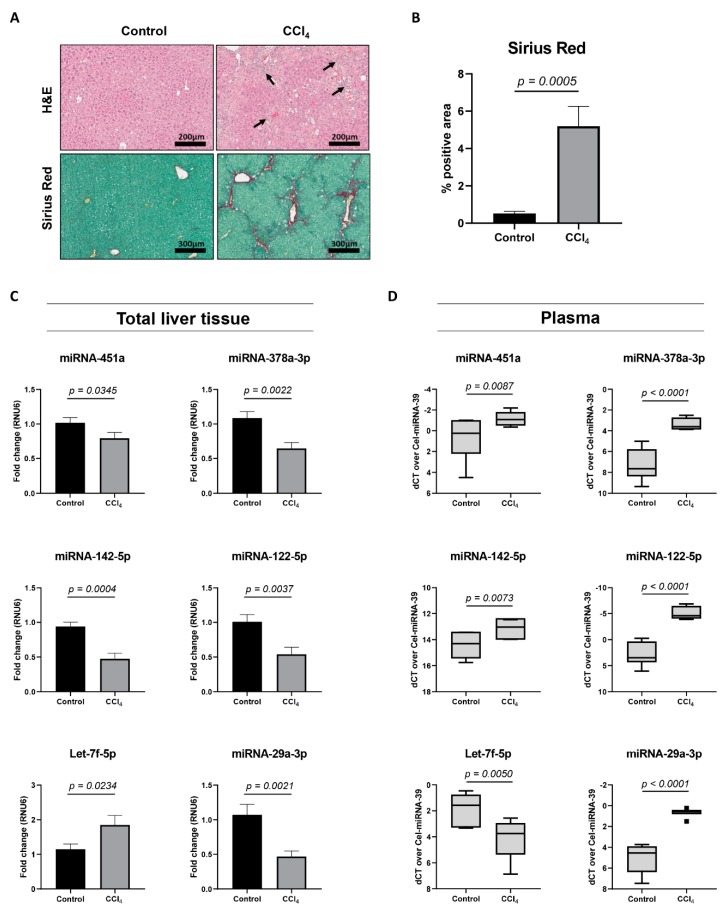
miRNA expression analysis in a CCl_4_-induced mouse model of liver fibrosis. (**A**) Total liver tissue of mice that received CCl_4_-injections two times a week, for a period of four weeks, and healthy controls, was used to visualize hepatocyte damage and inflammation (H&E), and cross-linked collagen deposition (Sirius Red). Arrows indicate infiltrated inflammatory cells. Representative images are shown. (**B**) The area of Sirius Red positive staining was calculated using Orbit analysis software and is plotted as percentage of the total area (*n* = 7 mice per group). (**C**) miRNA expression analysis of total liver tissue extracted from CCl_4_-injected mice, as compared to healthy controls (*n* = 7 mice per group). Results are shown as mean ± SEM. (**D**) Tukey boxplots represent miRNA expression values in plasma obtained from CCl_4_-injected mice, as compared to healthy controls (*n* = 7 mice per group). Obtained Ct levels were normalized by use of spiked-in Cel-miRNA-39. One-tailed unpaired t-test analysis was used to determine statistical significance.

Next, we analyzed the expression of miRNA-451a, miRNA-142-5p, Let-7f-5p, and miRNA-378a-3p in a well-studied mouse model of liver fibrosis, with repeated injections of carbon tetrachloride (CCl_4_) [20]. When mice are exposed to the CCl_4_-toxin two times a week, for four weeks, significant hepatocyte-damage and HSC-activation can be seen (Figure 3A,B). An analysis of total liver tissue from sick mice, compared to healthy controls, revealed significant changing levels for miRNA-451a, miRNA-142-5p, Let-7f-5p, and miRNA-378a-3p (Figure 3C), with overlapping expression patterns, as found in activating HSCs. The expression of two miRNAs extensively characterized in chronic liver diseases, miRNA-122-5p [21] and miRNA-29a-3p [22], was used as positive controls. Plasma obtained from the CCl_4_-mouse model identified significant changing expression levels of all analyzed miRNAs (Figure 3D). Interestingly, all miRNAs had a plasma expression pattern opposite to what was found in total liver tissue or activating HSCs. Altogether, these results suggest the potential use of these circulating miRNAs, without the need to distinguish between miRNAs packaged into extracellular vesicles or bound to (lipo-)proteins, as markers for HSC activation and fibrosis progression.

### 3.4. Patient Characteristics and Plasma miRNA Alterations During Liver Fibrosis Progression

Next, we investigated whether the plasma levels of these six miRNAs can be correlated to liver fibrosis severity in patients suffering from different chronic liver diseases. Patient characteristics are summarized in Table 1. A total of 208 patients were included, of which 92 patients were diagnosed with no or minimal fibrosis (F0–1) and 116 patients with significant fibrosis (F ≥ 2), as staged by elastography. Patients with various aetiologies of liver disease were recruited—chronic alcohol abuse (*n* = 33), chronic HBV/HCV infection (*n* = 74), and NAFLD (*n* = 101). Patients with chronic alcohol abuse or viral infection underwent transient elastography (FibroScan^®^) to distinguish significant liver fibrosis (F ≥ 2) from no or minimal fibrosis (F0–1); (median (25th; 75th percentile)) 12 (9.1; 33.6) kPa versus 5.2 (4.0; 6.1) kPa, respectively. Patients who presented with NAFLD all suffered from Diabetes Mellitus type 2 and underwent ARFI to distinguish significant liver fibrosis (F ≥ 2) from no or minimal fibrosis (F0–1); (median (25th; 75th percentile)) 1.525 (1.30; 1.68) m/s versus 1.15 (1.12; 1.20) m/s, respectively. Various clinical scoring algorithms, such as the AST/ALT ratio, Fib-4 score, APRI, and the recently developed PRTA-score [17], were calculated. All liver-related laboratory parameters, except for ALT and Creatinine values, and all fibrosis scoring tools are significantly different between the F0–1 and F ≥ 2 patient cohorts, validating the early- or late disease character of the included patients (Table 1).

**Figure 4:**
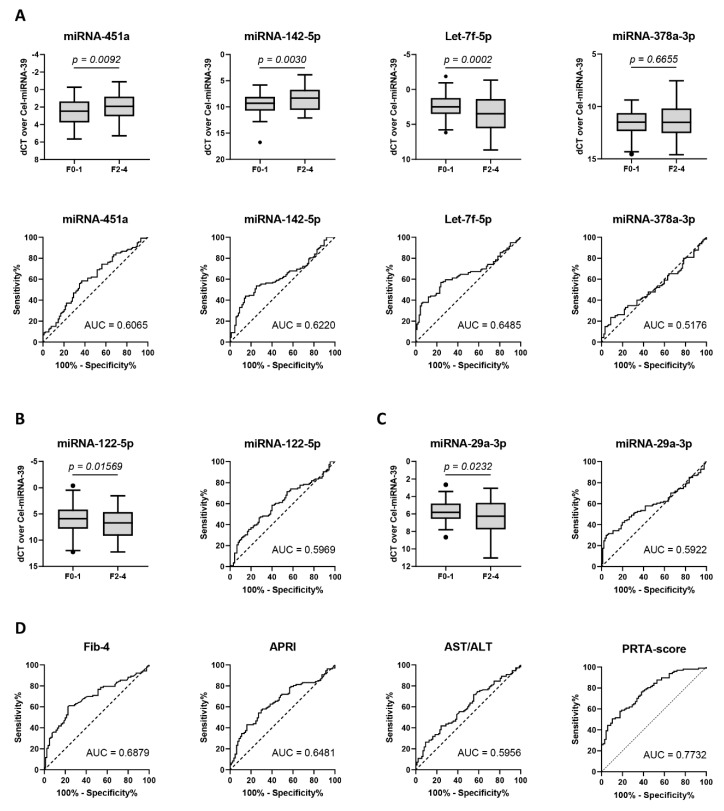
Evidence of significant liver fibrosis by the plasma levels of individual miRNAs. Expression analysis of circulating miRNAs in patients (*n* = 208) with chronic alcohol abuse, viral infection, and non-alcoholic fatty liver disease (NAFLD), with (F2–4) or without (F0–1) significant liver fibrosis. *p*-values were calculated using the Mann–Whitney U-test. Data is presented as Tukey boxplots. Receiver operating characteristic curve analysis was performed, and area under the curve (AUC) values were calculated to quantify the diagnostic value. The discriminative capacity of (**A**) candidate miRNAs was compared to (**B**) miRNA-122-5p and (**C**) miRNA-29a-3p and to (**D**) the serological scoring algorithms Fib-4, APRI, AST/ALT, and PRTA.

Analysis of the plasma of these patients showed that miRNA-451a and miRNA-142-5p were significantly up-regulated, while Let-7f-5p was significantly down-regulated, in patients with significant liver fibrosis (F ≥ 2). miRNA-378a-3p remained stable during fibrosis progression (Figure 4A). Area under receiver operating characteristic (AUROC) analysis identified comparable diagnostic utility among miRNA-451a (AUC = 0.6065), miRNA-142-5p (AUC = 0.6220), and Let-7f-5p (AUC = 0.6485) (Figure 4A and Supplementary Table S3). Late-stage fibrosis markers miRNA-122-5p (AUC = 0.5969) and miRNA-29a-3p (AUC = 0.5922) (Figure 4B,C and Supplementary Table S3) were found to have lower diagnostic utility. While APRI (AUC = 0.6481) and AST/ALT (AUC = 0.5956) were comparable to or were outperformed by the analyzed miRNAs, FIB-4 (AUC = 0.6879) and the PRTA-score (AUC = 0.7732) remained superior for the diagnosis of significant liver fibrosis (Figure 4D and Supplementary Table S3).

### 3.5. Association of Circulating miRNAs With Clinical Variables

We next examined the association between miRNA expression levels and clinical-pathological variables of the patient cohort. The levels of miRNA-451a (r = −0.2118), miRNA-142-5p (r = −0.2074), Let-7f-5p (r = 0.3426), miRNA-122-5p (r = 0.2193), and miRNA-29a-3p (r = 0.2413) were correlated with fibrosis severity, as determined by elastography (Supplementary Table S4 and Figure S2). Let-7f-5p was correlated with various fibrosis-linked parameters, such as decreasing albumin levels (r = −0.2031) and platelet counts (r = −0.3778), and to the fibrosis-scores Fib-4 (r = 0.3215), APRI (r = 0.2820), and PRTA-score (r = 0.4332), suggesting its association with hepatic fibrosis severity. As expected, miRNA-122-5p was strongly associated with AST (r = −0.2625) and ALT (r = −0.4093) levels, and thus marks hepatocyte damage. miRNA-142-5p (r = −0.1602), Let-7f-5p (r = 0.1416), and miRNA-122-5p (r = 0.2628) were associated with age, while Let-7f-5p (r = −0.1563) and miRNA-29a-3p (r = −0.2161) were associated with body mass index (Table 2).

### 3.6. Discrimination of Significant Liver Fibrosis by the miRFIB-Score

To investigate whether a combination of the evaluated miRNAs could be used to diagnose significant (F ≥ 2) liver fibrosis with a higher predictive value than the individual miRNAs, we created an miRNA-algorithm using logistic regression analysis. Here, the total patient cohort (*n* = 208) was randomly divided (Excel, Microsoft, WA, USA) into a derivation (*n* = 143) and validation (*n* = 65) cohort. Considering the lack of association between miRNA-378a-3p and fibrosis severity (Figure 3 and Supplementary Table S4), we chose to exclude this miRNA from the score. Combination of the other miRNA-variables generated the miRFIB-score:

miRFIB = 4.3799 + (0.70824 × Let-7f-5p (dCT)) − (0.090912 × miRNA-122-5p (dCT)) − (0.26149 × miRNA-142-5p (dCT)) − (0.53602 × miRNA-29a-3p (dCT)) − (0.041140 × miRNA-451a (dCT)).

**Figure 5:**
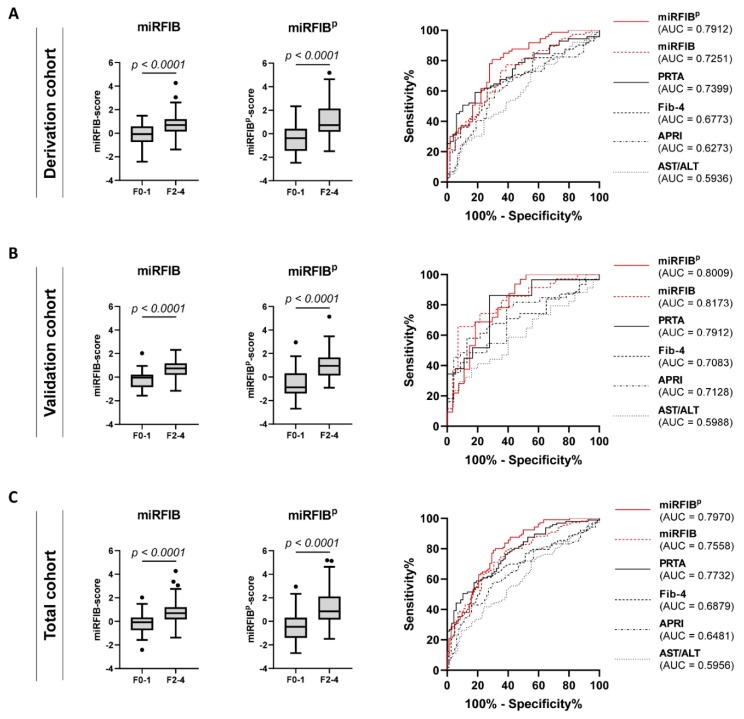
Diagnostic performance of the miRFIB- and the miRFIB^p^-score for significant liver fibrosis. Performance comparison of the miRFIB-, the miRFIB^p^-score, and commonly used validated diagnostic algorithms for the diagnosis of significant liver fibrosis (F2–4) in (**A**) the derivation, (**B**) validation, and (**C**) total patient cohort.

The diagnostic value of the miRFIB-score to diagnose significant liver fibrosis in the derivation cohort was superior (AUC = 0.7251) to the clinical scores AST/ALT, APRI, and Fib-4 (AUC of 0.5936, 0.6273, and 0.6773, respectively). The diagnostic value of the miRFIB-score was confirmed in the validation cohort (AUC = 0.8173) and total cohort (AUC = 0.7558) (Table 3 and Figure 5). Additionally, the miRFIB-score was found to be significantly correlated with fibrosis severity (r = 0.4365) (Supplementary Table S4) and was able to differentiate patients with specific stage F2 from patients with stage F0–1 (*p* < 0.0001) (Supplementary Figure S2).

### 3.7. Inclusion of PDGFRβ Improves the Diagnostic Power of the miRFIB-Score

We previously reported the diagnostic utility of circulating PDGFRβ protein levels to detect significant liver fibrosis [17]. Thus, we performed logistic regression analysis on the derivation cohort, combining PDGFRβ levels with our five-miRNA panel. This generated the miRFIB^p^-score, which was calculated as follows:

miRFIB^p^-score = (0.97229 × miRFIB-score) + (0.00021150 × PDGFRβ (pg/mL) − 1.8678.

The score had an increased diagnostic value for the identification of significant liver fibrosis in the derivation cohort (AUC = 0.7912; sensitivity = 80.82%; specificity = 70.37%), validation cohort (AUC = 0.8009; sensitivity = 68.75%; sensitivity = 81.48%), and total cohort (AUC = 0.7970; sensitivity = 79.05%; sensitivity = 69.51%) (Table 3 and Figure 5). The inclusion of PDGFRβ into the miRFIB-score further improved the correlation with fibrosis severity (r = 0.4847) (Supplementary Table S4), with a persistent possibility to differentiate patients with specific stage F2 liver fibrosis from patients with stage F0–1 fibrosis (*p* < 0.0001) (Supplementary Figure S2).

## 4. Discussion

Studies concerning the identification of novel non-invasive diagnostic tools suitable for the screening and monitoring of liver fibrosis in a general patient population remain limited. However, they are highly needed, as an accurate diagnosis of liver fibrosis has been shown to be a critical determinant of patient outcome [23,24]. Serological scoring tools, such as Fib-4, APRI, and AST/ALT, have been integrated into clinical practice, but lack accuracy for the identification of early stages of, and minor changes in, liver fibrosis [25,26,27]. Therefore, they are often only used as an indicator of the need for liver biopsy. The need for liver biopsy in current clinical practice thus remains. The identification of an adequate, sensitive, and specific serological marker for liver fibrosis would be of great value, as it could be used as an efficient first-line diagnostic step in screening at-risk patients [28], provide an easy tool for monitoring patients with fibrosis, and be of use in clinical trials evaluating fibrosis.

In the present study, we assessed the diagnostic utility of miRNAs differentially expressed during the activation of in vitro cultured primary HSCs, to identify significant liver fibrosis in a heterogeneous patient cohort with chronic viral infection, chronic alcohol abuse, and NAFLD. We identified an increased expression of miRNA-451a and miRNA-142-5p in the plasma of patients with significant liver fibrosis versus patients with no or mild fibrosis, whereas Let-7f-5p expression was decreased (Figure 4A). The observed down-regulation of miRNA-451a in total liver tissue of CCl_4_-injected mice (Figure 3C) mimics their down-regulated expression in livers of NASH patients, compared to patients with simple steatosis [29]. Additionally, its enhanced circulating expression, observed in our patient cohort with significant liver fibrosis (Figure 4A), was also seen when comparing the serum of NAFLD patients with healthy controls [30]. In contrast, one study identified a down-regulation of miRNA-451a in the plasma of cirrhotic HBV patients, compared to healthy controls [31]. These results could not be confirmed in our patient cohort with chronic viral infection. Differences in miRNA-451a expression between HBV and HCV patients should further be investigated. Su et al [32] identified the inflammatory signals IL4 and IL13 to increase miRNA-142-5p expression in macrophages, activating them towards a pro-fibrogenic character. Furthermore, they identify its up-regulation in liver tissue of six-weeks CCl_4_-treated C57BL/6J mice. This is in contrast to the down-regulation we observed in the liver tissue of four-weeks CCl_4_-treated Balb/c mice (Figure 3C). Differences in fibrosis progression or in miRNA expression between mouse strains could be the cause of such discrepancy. Our results concerning liver miRNA-142-5p expression seem to mimic the down-regulation seen in the liver tissue of cirrhotic HCV patients [33]. While decreasing levels of circulating Let-7a-5p, Let-7c-5p, and Let-7d-5p are correlated with fibrosis severity in patients with chronic HCV infection [34], the diagnostic utility of Let-7f-5p was not investigated in these studies. We show that circulating Let-7f-5p has the highest diagnostic value of all candidate miRNAs (Figure 4A and Supplementary Table S3) for the identification of significant liver fibrosis. Additionally, from the candidate miRNA panel, Let-7f-5p was the only miRNA to be correlated with the Fib-4, APRI, and PRTA-scoring tools (Table 2), what further underlines its diagnostic potential.

In the search for the ideal diagnostic miRNA-based algorithm, we supplemented our candidate miRNA panel with the well-studied miRNA-122-5p and miRNA-29a-3p. miRNA-122-5p is the most abundant miRNA in the liver, with dominant expression in the hepatocytes (Supplementary Figure S3) [35], where it is involved in cholesterol synthesis [36]. The elevated levels of circulating miRNA-122-5p in patients with early stage fibrosis (F1–2) as compared to healthy controls are suggested to represent miRNA-release from injured hepatocytes. On the other hand, the decreased circulating miRNA-122-5p levels during later stages of fibrosis (F3–4) would be caused by the progressive loss of functional hepatocytes in the injured liver [37]. Its diagnostic utility for late-stage liver fibrosis and cirrhosis has already been suggested in various liver disease aetiologies [38,39,40,41,42,43]. Our study shows that while miRNA-122-5p has little diagnostic value on its own for significant fibrosis (Figure 4B), its contribution is essential to the miRFIB-score (Figure 5). In contrast to the hepatocyte-specificity of miRNA-122-5p, miRNA-29a-3p shows the highest expression in HSCs (Supplementary Figure S3), undergoing down-regulation upon in vitro and in vivo activation [22]. Its potential use as a therapeutic target has been elaboratively reported by the group of Y.H. Huang et al. using various mouse models of liver disease [44,45,46,47]. The negative correlation of circulating miRNA-29a-3p levels with fibrosis/cirrhosis severity has been shown in patients with NAFLD [48], HBV [49], HCV, alcohol abuse, and biliary disease [22].

Individually, all of the analyzed significantly dysregulated miRNAs had low predictive values for the diagnosis of significant liver fibrosis, with AUC values ranging from 0.59 to 0.64 (Figure 4A–C and Supplementary Table S3). However, using logistic regression analysis, we generated an algorithm, the miRFIB-score, consisting of miRNA-142-5p, miRNA-451a, Let-7f-5p, miRNA-122-5p, and miRNA-29a-3p, with a predictive value superior to the clinical scoring systems Fib-4, APRI, and AST/ALT (Figure 5 and Table 3). As we recently reported the highly discriminative potential of circulating PDGFRβ-levels for significant liver fibrosis in patients with various aetiologies of liver disease [17], we generated a second diagnostic algorithm combining the miRFIB-score with such circulating PDGFRβ-levels, the miRFIB^p^-score. A marked improvement in diagnostic values was observed for this combinatory algorithm (Figure 5 and Table 3).

An unexpected finding was the discrepancy between the enhanced expression of miRNA-122-5p and miRNA-29a-3p in the plasma of mice treated with CCl_4_ (Figure 3D), and their lowered expression levels in the plasma of patients with significant fibrosis (Figure 4C). All other tested miRNAs seem to have an overlapping expression pattern between mouse and human subjects. In our experiments, Balb/c mice underwent only four weeks of CCl_4_-injections, which is thought to represent early-stage fibrosis. To induce late-stage fibrosis, or cirrhosis, 8–20 weeks of CCl_4_-injections should be performed [20]. However, the enhanced expression of miRNA-122-5p in the plasma of CCl_4_-injected mice is in line with our previous results comparing the plasma of patients with early-stage fibrosis to healthy subjects [14]. Alternatively, the enhanced expression of miRNA-122-5p in the plasma of mice treated with CCl_4_ could reflect the hepatocyte damage caused by the last CCl_4_-injection, since samples were taken only 24 h later. For miRNA-29a-3p, this is likely not the case, since this miRNA has thus far not been associated with hepatocyte damage. To further investigate this, healthy individuals should be included in future studies.

miRNA-451a, miRNA-142a-5p, Let-7f-5p, and miRNA-378a-3p were found to be significantly dysregulated in activated HSCs, as compared to quiescent controls (Figure 1D), which suggests a role in the HSC activation process. To further investigate this, target prediction was performed, and we focused on the target genes regulated by all four HSC-activation linked miRNAs. Of the predicted 13 overlapping miRNA targets (Figure 2A), three genes (*Ankrd52*, *Clcn5*, and *Peg10*), were found to be significantly up-regulated upon HSC activation (Figure 2C). As miRNAs negatively regulate gene expression by mRNA decay or inhibition of translation [50], and due to the dominantly down-regulated expression of the selected miRNAs, we hypothesize *Ankrd52*, *Clcn5*, and *Peg10* to be regulated by the selected miRNAs. However, to confirm this, further functional studies should be performed. While the roles of *Ankrd52* and *Clcn5* in liver disease remain unclear, *Peg10* has been widely studied in hepatocellular carcinoma (HCC) pathology. More specifically, *Peg10* expression levels are elevated in HCC [51], where it is found to inhibit the pro-apoptotic mediator *Siah1* [52], and stimulate cell proliferation by association with c-MYC [53]. Additionally, *Peg10* tissue mRNA levels mark HCC progression and poor survival [54,55]. Due to its high expression in activated HSCs (Figure 2C), and its important functionality in HCC, it would be of interest to investigate its role in liver fibrogenesis. As it cannot be excluded that a target gene is dominantly regulated by just one specific miRNA, it is possible that the increasing Let-7f-5p levels found upon HSC activation lead to the identified down-regulation of multiple predicted target genes (Figure 2D). Among these, *Cpeb3* and *Gnai3* have proven functionality as tumor suppressors in HCC. Both genes are found to undergo negative regulation by miRNAs, *Cpeb3* by miRNA-452-3p and miRNA-107 [56,57], and *Gnai3* by miRNA-222 [58]. However, their role during liver fibrosis remains to be determined.

Whether a miRFIB- or miRFIB^p^-score can be integrated into the clinical practice will depend on future technical developments. Currently, a combination of protein and miRNA detection from the same plasma sample is not standard practice in hospital settings. Due to this more complex character, we expect the miRFIB^p^-score to have a more important financial cost, compared to the miRFIB-score. However, our analysis identified the miRFIB^p^-score to have superior diagnostic value for the identification of significant liver fibrosis and an in-depth cost–benefit analysis should determine if this diagnostic superiority outweighs the additional costs. Furthermore, the manipulation of blood samples should be performed with great care, since hemolysis of red blood cells may result in the release of their cytoplasmic miRNAs, such as miRNA-451a [59,60], and thus influence the results of the miRNA-based diagnostic algorithms. Additionally, the time interval between blood sampling and plasma storage should be kept as short as possible, as specific miRNAs, including miRNA-122-5p, can undergo a time-dependent decline in stability when the sample is kept at room temperature [61].

There were a number of limitations with the current study. The patient cohort size was relatively small and contained an imbalance in the presence of liver disease aetiologies. Although recent research has reported substantial differences in miRNA expression values in the plasma of patients with HBV infection versus patients with HCV infection [62], due to insufficient patient numbers we were unable to make any claims regarding such differences in our patient cohort. Furthermore, due to the cross-sectional character of the study, we did not possess any clinical follow-up material of the included patients. We were thus unable to test the prognostic ability of the miRFIB- and miRFIB^p^-score. Finally, all included patients were staged for liver fibrosis by use of elastography. Future studies should focus on the validation of our results using plasma obtained from biopsy-staged patients. Moreover, the score should be tested during treatment to study if the scores can be used to evaluate early changes in fibrosis, and possibly predict the outcome.

In conclusion, we have identified five miRNAs that, when combined into the predictive miRFIB-signature, had high diagnostic values for significant liver fibrosis in a heterogeneous patient population with chronic alcohol abuse, viral infection, and NAFLD. Combining the miRFIB-score with circulating PDGFRβ-levels increased its diagnostic utility. Although these proposed scores require further validation, they may provide crucial information regarding liver fibrosis severity and evolution. Thanks to their non-invasive character, the scores would allow repeated measures and objective interpretation, at a relatively low financial cost. Additionally, functional studies could unravel the importance of the selected miRNAs during fibrogenesis and fibrolysis, and their potential utility as therapeutic targets.

## Figures and Tables

**Table 1:** Baseline characteristics of the patient cohort.

Patient Cohorts	F0–1	F2–4	*p* Value
Individuals, *n*	92	116	
			
**Disease aetiology: *n* (%)**			
Alcoholic liver disease	6 (7%)	27 (23%)	
Viral liver disease	46 (50%)	28 (24%)	
NAFLD	40 (43%)	61 (53%)	
			
**Characteristics**			
Age (years): median (IQR)	52 (42–63)	57 (51–65)	0.0003
Male, *n* (%)	52 (57%)	84 (72%)	0.0267
BMI (kg/m^2^): median (IQR)	27.44 (24.16–32.35)	29.96 (25.18–34.23)	ns
			
**Laboratory parameters: median (IQR)**			
AST (IU/L)	34 (24–48)	40 (28–67)	0.0048
ALT (IU/L)	43 (34–62)	48 (32–71)	ns
Alk Phos (IU/L)	68 (54–87)	87 (66–130)	<0.0001
GGT (IU/L)	39 (23–83)	71 (40–154)	<0.0001
Total bilirubin (mg/dL)	0.60 (0.47–0.79)	0.76 (0.57–1.20)	0.0005
Albumin (g/L)	43 (41–46)	42 (39–45)	0.0035
Thrombocytes (×10^3^/mm^3^)	231 (202–277)	202 (149–255)	0.0006
Creatinine (mg/dL)	0.85 (0.70–1.02)	0.87 (0.74–1.04)	ns
			
**Fibrosis scoring: median (IQR)**			
AST/ALT ratio	0.76 (0.62–0.87)	0.82 (0.67–1.18)	0.0211
APRI	0.35 (0.24–0.57)	0.56 (0.32–0.88)	0.0005
Fib-4	1.13 (0.83–1.49)	1.67 (1.08–2.80)	<0.0001
PRTA-score	7.18 (4.49–9.87)	11.63 (7.95–20.40)	<0.0001

n: number; NAFLD: non-alcoholic fatty liver disease; IQR: interquartile range; BMI: body mass index; AST: aspartate aminotransferase; ALT: alanine aminotransferase; Alk Phos: alkaline phosphatase; GGT: gamma-glutamyl transferase; AST/ALT ratio: aspartate aminotransferase/alanine aminotransferase ratio; APRI: AST to platelet ratio index; Fib-4: Fibrosis-4; PRTA-score: PDGFRβ-thrombocytes-albumin score; ns: not significant.

**Table 2:** Correlations of circulating miRNA expression levels with clinical parameters.

	miRNA-451a		miRNA-142-5p		Let-7f-5p		miRNA-378a-3p		miRNA-122-5p		miRNA-29a-3p	
	*r*	*p*	*r*	*p*	*r*	*p*	*r*	*p*	*r*	*p*	*r*	*p*
**Age**	−0.0409	ns	−0.1602	0.0234	0.1416	0.0413	0.0905	ns	0.2628	0.0001	0.0096	ns
**BMI**	0.0280	ns	−0.0397	ns	−0.1563	0.0287	−0.1037	ns	−0.0667	ns	−0.2161	0.0025
**AST**	−0.1554	0.0318	0.0076	ns	0.1371	ns	−0.1038	ns	−0.2625	0.0002	0.1150	ns
**ALT**	−0.0727	ns	0.0207	ns	0.0534	ns	−0.1412	0.0484	−0.4093	<0.0001	−0.0166	ns
**Alk Phos**	−0.0022	ns	−0.0804	ns	0.2027	0.0050	−0.0047	ns	0.2059	0.0046	0.1600	0.0287
**GGT**	−0.1372	ns	−0.1756	0.0174	0.1730	0.0163	−0.0621	ns	0.0060	ns	0.0899	ns
**Bilirubin**	−0.0609	ns	−0.0237	ns	0.3194	<0.0001	0.1295	ns	0.1213	ns	0.2790	0.0001
**Albumin**	−0.1064	ns	0.0365	ns	−0.2031	0.0067	−0.0148	ns	−0.2394	0.0014	−0.1747	0.0207
**Platelet count**	0.1243	ns	−0.1139	ns	−0.3778	<0.0001	−0.1629	0.0255	−0.1050	ns	−0.3514	<0.0001
**Creatinine**	−0.0419	ns	−0.0399	ns	0.0054	ns	−0.0094	ns	0.0652	ns	−0.0257	ns
**AST/ALT**	−0.1026	ns	−0.0126	ns	0.0756	ns	0.0311	ns	0.1920	0.0072	0.1627	0.0234
**Fib-4**	−0.1223	ns	0.0439	ns	0.3215	<0.0001	0.1400	ns	0.0607	ns	0.2852	0.0001
**APRI**	−0.1551	0.0365	0.1039	ns	0.2820	<0.0001	0.0321	ns	−0.1573	0.0321	0.2551	0.0005
**PRTA-score**	−0.0559	ns	0.0266	ns	0.4332	<0.0001	0.1479	ns	0.2401	0.0020	0.3447	<0.0001

Correlations were evaluated by the Pearson’s correlation coefficient (r). ns: not significant; BMI: body mass index; AST: aspartate aminotransferase; ALT: alanine aminotransferase; Alk Phos: alkaline phosphatase; GGT: gamma-glutamyl transferase; AST/ALT ratio: aspartate aminotransferase/alanine aminotransferase ratio; APRI: AST to platelet ratio index; Fib-4: Fibrosis-4; PRTA-score: PDGFRβ-thrombocytes-albumin score.

**Table 3:** Performance of the miRFIB- and miRFIB^p^-score, as compared to the AST/ALT, APRI, Fib-4, and PRTA scoring algorithms, for the detection of significant liver fibrosis (F ≥ 2).

	AUC	95% CI	Optimal Cut-Off	Sensitivity (%)	Specificity (%)	PPV	NPV
**AST/ALT**							
Derivation	0.5936	0.4986–0.6886	0.6948	73.68	45.16	62.87	58.65
Validation	0.5988	0.4546–0.7430	1.025	38.24	84.00	75.08	51.90
Total	0.5956	0.5166–0.6747	0.8725	41.82	75.86	68.59	50.85
							
**APRI**							
Derivation	0.6273	0.5313–0.7234	0.4928	59.46	68.97	70.72	57.44
Validation	0.7128	0.5773–0.8482	0.7531	45.45	95.65	92.94	58.18
Total	0.6481	0.5696–0.7267	0.4928	57.01	70.37	70.80	56.49
							
**Fib-4**							
Derivation	0.6773	0.5847–0.7698	1.505	61.11	73.68	74.53	60.05
Validation	0.7083	0.5692–0.8474	1.520	58.06	86.96	84.88	62.19
Total	0.6879	0.6112–0.7647	1.505	60.19	77.50	77.13	60.70
							
**PRTA-score**							
Derivation	0.7399	0.6525–0.8272	10.36	59.15	81.63	80.23	61.32
Validation	0.7912	0.6566–0.9258	7.842	86.21	72.22	79.64	80.60
Total	0.7732	0.7033–0.8431	11.59	50.52	89.71	86.09	58.99
							
**miRFIB**							
Derivation	0.7251	0.6393–0.8110	0.1109	77.33	61.40	71.63	68.24
Validation	0.8173	0.7112–0.9235	0.3412	65.71	92.86	92.06	68.24
Total	0.7558	0.6887–0.8229	0.1404	78.38	62.35	72.41	69.59
							
**miRFIB^p^**							
Derivation	0.7912	0.7120–0.8704	0.0673	80.82	70.37	77.47	74.43
Validation	0.8009	0.6844–0.9175	0.3840	68.75	81.48	82.39	67.41
Total	0.7970	0.7329–0.8611	0.1043	79.05	69.51	76.57	72.47

AST/ALT ratio: aspartate aminotransferase/alanine aminotransferase ratio; APRI: AST to platelet ratio index; Fib-4: Fibrosis-4; PRTA-score: PDGFRβ-thrombocytes-albumin score; AUC: area under the curve; CI: confidence interval; PPV: positive predictive value; NPV: negative predictive value.

## Supplementary Materials

The following are available online at https://www.mdpi.com/2073-4409/8/9/1003/s1; **Supplementary Materials and Methods**: Extracellular vesicle isolation and RNA extraction, Nanostring miRNA analysis, Simultaneous isolation of different liver cell types. **Supplementary Figures and Tables**: Figure S1: Top enriched miRNAs in EVs derived from activating HSCs, Figure S2: Fibrosis-stage specific presence of circulating miRNAs, Figure S3: miRNA expression analysis in liver cell types, Table S1: miRNA primers, Table S2: mRNA primers, Table S3: Performance of individual plasma miRNAs, as compared to the AST/ALT, APRI, Fib-4, and PRTA scoring algorithms, for the detection of significant liver fibrosis (F ≥ 2), Table S4: Correlation of circulating miRNA expression levels with fibrosis stage.
