# Supplementary material for: The miRFIB-Score: A Serological miRNA-Based Scoring Algorithm for the Diagnosis of Significant Liver Fibrosis
Source: Cells. 2019 Aug 29;8(9):1003. doi: 10.3390/cells8091003 (PMC6770498; doi:10.3390/cells8091003)
Supplement: Supplementary file 1 [file cells-08-01003-s001.pdf]

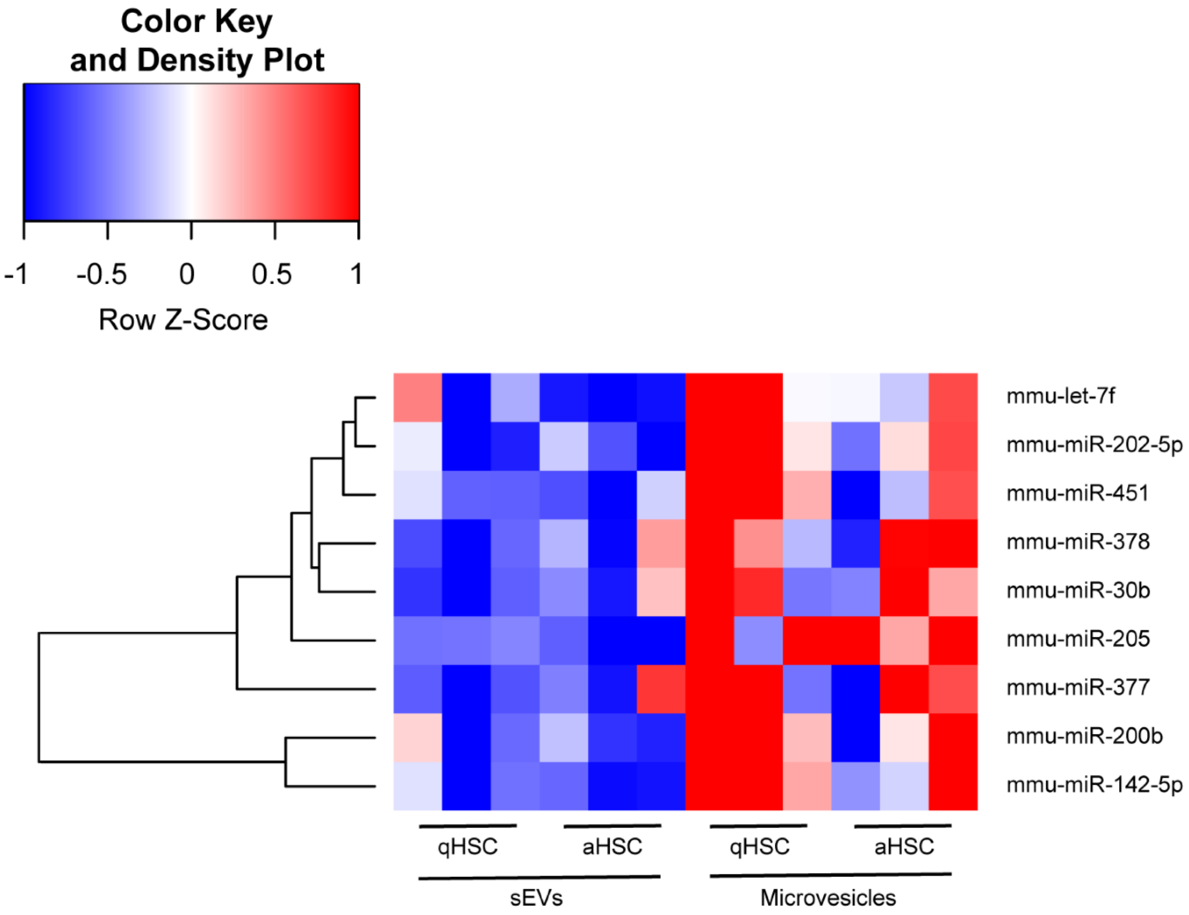

Figure S1. Top enriched miRNAs in extracellular vesicles (EVs) derived from activating HSCs. Heatmap visualizing miRNA expression levels of the top-enriched miRNAs in EVs, both small EVs (sEVs) and microvesicles, obtained from the conditioned medium of quiescent (2 days in culture) and activated (10 days in culture) primary mouse HSCs.

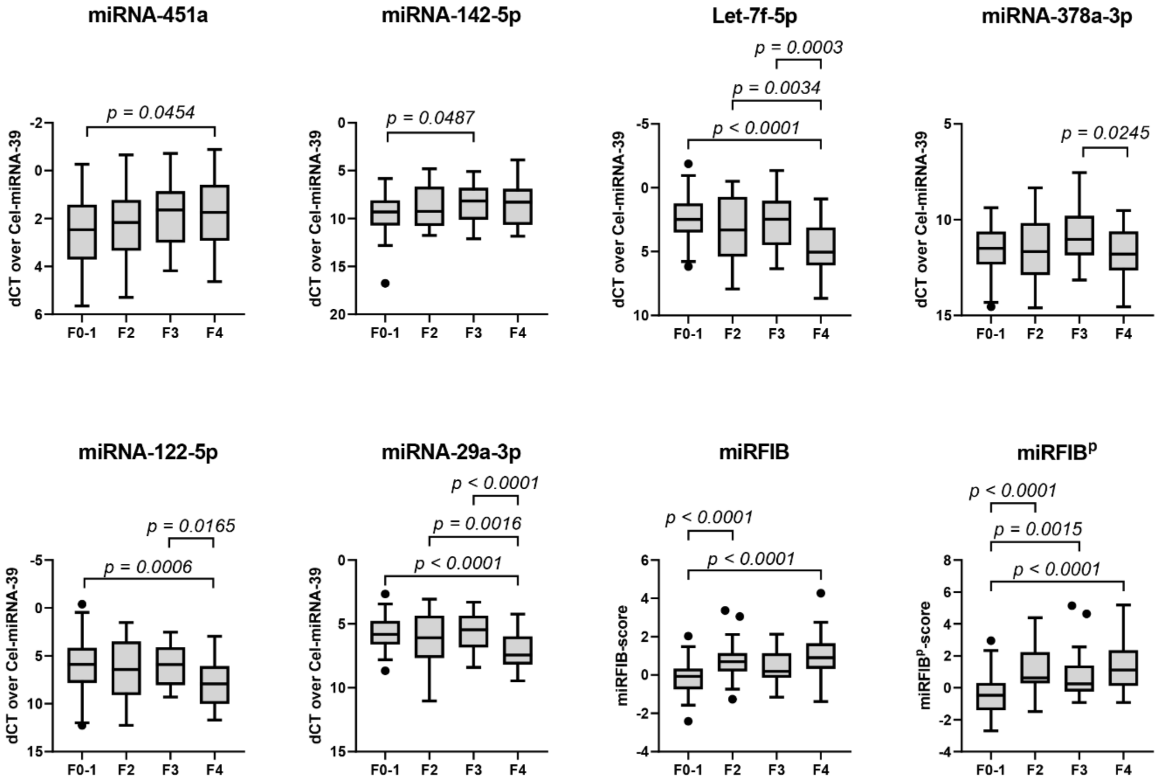

Figure S2. Fibrosis-stage specific presence of circulating miRNAs. The total patient cohort was divided based on the elastography-proven stage of liver fibrosis. Significant differences in expression levels between the various stages of liver fibrosis were determined using the Kruskal-Wallis test with Dunn's multiple comparisons test. Data is presented as Tukey boxplots.

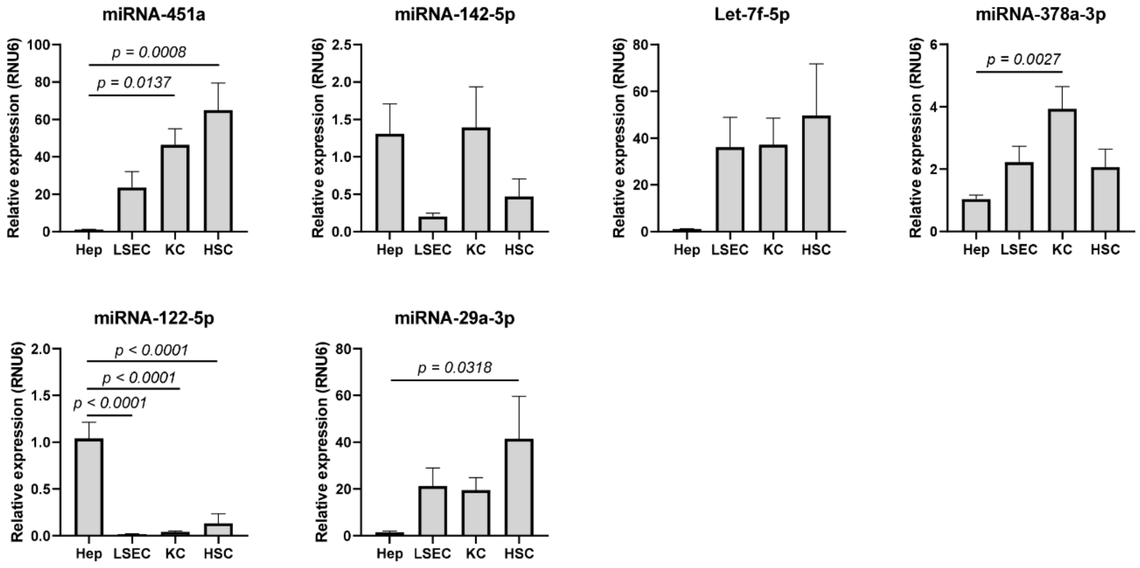

Figure 3. miRNA expression analysis in liver cell types. miRNA expression levels were determined by use of qPCR, and compared between hepatocytes and liver sinusoidal endothelial cells, Kupffer cells and HSCs. Statistical significance was determined using one-way ANOVA with Dunnett's multiple comparison test. Results are shown as mean ± SEM; $n = 6$.

Table 1. miRNA primers.

	Accession number		Primer sequence (5′-3′)
	Mouse	Human	
miRNA-122-5p	MIMAT0000246	MIMAT0000421	TGGAGTGTGACAATGGTGTTG
miRNA-29a-3p	MIMAT0000535	MIMAT0000086	TAGCACCATCTGAAATCGGTTA
miRNA-202-5p	MIMAT0004546	MIMAT0002810	TTCCTATGCATATACTTCTT
miRNA-30b-5p	MIMAT0000130	MIMAT0000420	TGTAAACATCCTACACTCAGCT
miRNA-205-5p	MIMAT0000238	MIMAT0000266	TCCTTCATTCCACCGGAGTCTG
miRNA-451a	MIMAT0001632	MIMAT0001631	AAACCGTTACCATTACTGAGTT
miRNA-142-5p	MIMAT0000154	MIMAT0000433	CATAAAGTAGAAAGCACTACT
Let-7f-5p	MIMAT0000525	MIMAT0000067	TGAGGTAGTAGATTGTATAGTT
miRNA-378a-3p	MIMAT0003151	MIMAT0000732	ACTGGACTTGGAGTCAGAAGG
miRNA-200b-3p	MIMAT0000233	MIMAT0000318	TAATACTGCCTGGTAATGATGA
miRNA-377-3p	MIMAT0000741	MIMAT0000730	ATCACACAAAGGCAACTTTTGT
RNU6	/	/	ACGCAAATTCGTGAAGCGTT
Cel-miRNA-39-3p	/	/	AGCTGATTTCGTCTTGGTAATA

Table 2. mRNA primers.

	Forward primer<br>Primer sequence (5′-3′)	Revers primer<br>Primer sequence (5′-3′)
Peg10	TGCTTGCACAGAGCTACAGTC	AGTTTGGGATAGGGGCTGCT
Surf4	ATGGGACAGAACGACCTGATG	GGTGTCGATATAGTCACGCTG
Aw549877	GGCTCACATACAGCACCTTAG	AGTCCTCTGGAAAATCCTCATCT
Zfp516	ACCGGACAGGAACTCTGATTC	GAGGTGCTCTTAGTAGGGCTG
Ankrd52	CCAGGCCATCTTTAGCCGAG	ATGCAATGGGGTTCGCCTC
Atxn713	TTGTCTGGCCTGGATAACAGC	CCGGTGTACTTCAAAGCAGAATC
Ppp3r1	GAAGGAGTGTCTCAGTTCAGTG	ACGAAAAGCAAACCTCAACTTCT
Aff4	ATGAACCGTGAAGACCGGAAT	TGCTAGTGACTTTGTATGGCTCA
Dnajc27	ACGCCGAAGTGGGGAAAAG	GAAGAAGGGATGTCCAGCCAT
Ctsb	TCCTTGATCCTTCTTTCTTGCC	ACAGTGCCACACAGCTTCTTC
Clcn5	GAGGAGCCAATCCCTGGTGTA	TTGGTAATCTCTCGGTGCCTA
Cpeb3	ATCTCGCCGCTCAAAAAGC	GGAAAGCGTTATCCTCCATCCA
Gnai3	CCAGACCAACTACATTCCAACTC	AATTGCTGTCACTCCCTCAAAA
Acta2	CCAGCACCATGAAGATCAAG	TGGAAGGTAGACAGCGAAGC
Col1a1	ACCTAAGGGTACCGCTGGA	ACCTAAGGGTACCGCTGGA
Gapdh	TCGAGATCGCCACCTACAG	GTCTGTACAGGAATGGTGATGC
Lox	CTCCTGGGAGTGGCACAG	CTTGCTTTGTGGCCTTCAG

Table 3. Performance of individual plasma miRNAs, as compared to the AST/ALT, APRI, Fib-4, and PRTA scoring algorithms, for the detection of significant liver fibrosis (F ≥ 2).

	AUC	95% CI	Optimal cut-off	Sensitivity (%)	Specificity (%)	PPV	NPV
**miRNA-451a**	0.6065	0.5282-0.6484	2.224	58.41	62.92	66.50	54.55
**miRNA-142-5p**	0.6220	0.5445-0.6994	7.653	43.75	85.23	78.87	54.59
**Let-7f-5p**	0.6485	0.5739-0.7231	3.292	56.90	76.09	74.99	58.35
**miRNA-378a-3p**	0.5176	0.4389-0.5962	10.27	23.48	91.30	77.28	48.63
**miRNA-122-5p**	0.5969	0.5195-0.6744	7.102	46.55	72.22	67.87	51.74
**miRNA-29a-3p**	0.5922	0.5147-0.6698	7.399	29.82	95.60	89.52	51.94
**Fib-4**	0.6879	0.6112-0.7647	1.505	60.19	77.50	77.13	60.70
**APRI**	0.6481	0.5696-0.7267	0.4928	57.01	70.37	70.80	56.49
**AST/ALT**	0.5956	0.5166-0.6746	0.8725	41.82	75.86	68.59	50.85
**PRTA-score**	0.7732	0.7033-0.8431	11.59	50.52	89.71	86.09	58.99

AST/ALT ratio: aspartate aminotransferase/alanine aminotransferase ratio; APRI: AST to platelet ratio index; Fib-4: Fibrosis-4; PRTA-score: PDGFRβ-thrombocytes-albumin score; AUC: area under the curve; CI: confidence interval; PPV: positive predictive value; NPV: negative predictive value.

Table 4. Correlation of circulating miRNA expression levels with fibrosis stage.

	Correlation to F-score	
	r	*p*
**miRNA-451a**	−0.2118	0.0025
**miRNA-142-5p**	−0.2074	0.0032
**Let-7f-5p**	0.3426	<0.0001
**miRNA-378a-3p**	−0.0119	ns
**miRNA-122-5p**	0.2193	0.0015
**miRNA-29a-3p**	0.2413	0.0005
**miRFIB**	0.4365	<0.0001
**miRFIB^{P}**	0.4847	<0.0001

Correlations were evaluated by the Pearson's correlation coefficient (r). ns: not significant.

Supplementary Materials and Methods

Extracellular vesicle isolation and RNA extraction

Primary mouse quiescent HSCs were cultured in Dulbecco's modified Eagle's medium (Lonza, Verviers, Belgium) supplemented with 10% exosome-depleted foetal bovine serum (Lonza, Verviers, Belgium), 2 mM L-glutamine (Ultraglutamine 1®) (Lonza), 100 U/mL penicillin and 100 µg/mL streptomycin (Pen-Strep®) (Lonza). Conditioned medium was collected after 2 days or 10 days of culture. New medium was added every 2 days. Cellular debris was removed by centrifugation at 300 g for 5 min (4 °C) and 2500 g for 20 min (4 °C). Microvesicles were pelleted by centrifugation at 10,000 g for 30 min (4 °C). The supernatant was further centrifuged at 100,000 g for 2 h (4 °C) to pellet small extracellular vesicles (sEVs), which were then washed once by resuspension in PBS, followed by a final centrifugation step at 100,000 g for 2 h (4 °C). Microvesicle and sEV pellets were resuspended in a small volume of PBS. Purity of the vesicle suspensions were verified by analysis of size using the ZetaView® PMX110 (Particle Metrix, Meerbusch, Germany) and presence of specific vesicle markers using western blot, as shown previously [1].

The obtained microvesicle and sEV suspensions were depleted from contaminating proteins by incubation with 0.5 mg proteinase K (ThermoFisher scientific) for 30 min at 55 °C. Total RNA was extracted by use of the Quick RNA miniprep (Zymo Research, CA, USA). Synthetic spike-in ath-miRNA-159a, cel-miRNA-248 and osa-miRNA-414 were added to the vesicle lysates before proceeding with the manufacturer's protocol.

Nanostring miRNA analysis

The RNA samples obtained from extracellular vesicles (microvesicles and sEVs) extracted from qHSCs (day 2) and aHSC (day 10) were submitted to the BRIGHTcore facility of the Vrije Universiteit Brussel (VUB) for further processing by the NanoString nCounter system (NanoString, Washington, USA). The nCounter Mouse v1.5 miRNA panel was used, which can analyse the expression of up to 578 endogenous miRNAs. Raw counts were obtained and analysed using the nSolver software. The background threshold was determined using the geometric mean of negative control counts. For technical variations, normalization was based on the geometric mean of the spike-in ath-miRNA-159a, cel-miRNA-248 and osa-miRNA-414. Normalized counts were imported into RStudio (https://www.rstudio.com) and selected highly expressed miRNAs (sum of all counts per miRNA > 800). These miRNA expression values were scaled per miRNA and visualized using heatmap with R package "gplots".

miRNA targets were predicted using TargetScan (http://www.targetscan.org/), miRDB (http://www.mirdb.org/), starBase (http://starbase.sysu.edu.cn/), and miRTarBase (http://mirtarbase.mbc.nctu.edu.tw/). Target lists were imported into RStudio, merged together and visualized using Venn diagram with R package "gplots".

Simultaneous isolation of different liver cell types

Liver cell populations were isolated based on the expression of cell-type specific markers, as described earlier [2]. In summary, murine liver were digested using enzymatic solutions consisting of collagenase (Roche diagnostics, Mannheim, Germany) and pronase E (Merck, Darmstadt, Germany). The resulting cell suspension was used in low-speed centrifugation steps to separate the non-parenchymal fraction from the hepatocytes. Next, the non-parenchymal fraction (NPF) was incubated with anti-F4/80-APC (MF8021, Thermo Scientific, USA) and anti-CD32-PE (ab30357, Abcam, UK). NPF was then analysed with FACS (FACS Aria II, Becton-Dickinson, Belgium) and used to isolate liver sinusoidal endothelial cells (LSEC, CD32+F4/80-UV-), Kupffer cells (CD32-F4/80+UV-) and HSCs (CD32-F4/80-UV+).

References

1. Lambrecht, J.; Verhulst, S.; Mannaerts, I.; Sowa, J.P.; Best, J.; Canbay, A.; Reynaert, H.; van Grunsven, L.A. A PDGFRbeta-based score predicts significant liver fibrosis in patients with chronic alcohol abuse, NAFLD and viral liver disease. *EBioMedicine* **2019**, *43*, 501–512, doi:10.1016/j.ebiom.2019.04.036.
2. Stradiot, L.; Verhulst, S.; Roosens, T.; Oie, C.I.; Moya, I.M.; Halder, G.; Mannaerts, I.; van Grunsven, L.A. Functionality based method for simultaneous isolation of rodent hepatic sinusoidal cells. *Biomaterials* **2017**, *139*, 91–101, doi:10.1016/j.biomaterials.2017.05.047.
